# *CELLOPT*: improved unit-cell parameters for electron diffraction data of small-molecule crystals

**DOI:** 10.1107/S160057672200276X

**Published:** 2022-04-29

**Authors:** Tim Gruene, Max T. B. Clabbers, Jens Luebben, Jia Min Chin, Michael R. Reithofer, Frank Stowasser, André M. Alker

**Affiliations:** a Institute of Inorganic Chemistry, Faculty of Chemistry, University of Vienna, Austria; b Department of Materials and Environmental Chemistry, Stockholm University, Sweden; c Bruker AXS, Germany; d Institute of Inorganic Chemistry – Functional Materials, Faculty of Chemistry, University of Vienna, Austria; e Roche Pharma Research and Early Development, Basel, Switzerland

**Keywords:** electron diffraction, precision of unit-cell parameters, crystal structure determination, compensation for experimental and instrumental errors

## Abstract

Electron diffraction enables structure determination of organic small molecules using crystals that are too small for conventional X-ray crystallography. However, because of uncertainties in the experimental parameters, notably the detector distance, the unit-cell parameters and the geometry of the structural models are typically less accurate and precise compared with results obtained by X-ray diffraction. Here, an iterative procedure to optimize the unit-cell parameters obtained from electron diffraction using idealized restraints is proposed. The cell optimization routine has been implemented as part of the structure refinement, and a gradual improvement in lattice parameters and data quality is demonstrated. It is shown that cell optimization, optionally combined with geometrical corrections for any apparent detector distortions, benefits refinement of electron diffraction data in small-molecule crystallography and leads to more accurate structural models.

## Abstract 2

Iterative optimization of the unit-cell parameters improves the refinement of organic small-molecule structures using electron diffraction data.

## 1. Introduction

A crystallographic diffraction experiment aims to determine the unit-cell constants *a*, *b*, *c*, α, β, γ and the reflection intensities. During data reduction, the unit-cell constants are related to the diffraction pattern via the Laue equations: they serve to predict the spot positions on the detector surface. The parameters describing the geometry of the experiment can be refined during data integration: detector distance, unit-cell constants, beam direction, beam divergence, rotation axis and possibly more, depending on the data reduction program. In X-ray crystallography, the refinement of these parameters results in standard uncertainties for the unit-cell parameters in the range of 0.001–0.01 Å for the cell lengths and 0.001–0.01° for the angles.

In electron diffraction, the standard uncertainties of the unit-cell parameters of organic and inorganic small molecules are typically higher (Dorset, 1995 ▸; Mugnaioli *et al.*, 2009 ▸; Ångström *et al.*, 2018 ▸; Wang *et al.*, 2018 ▸; Clabbers *et al.*, 2019 ▸). Since the unit cell is used to calculate bond distances and bond angles, the stereochemistry of the molecules is often poorly defined compared with average values. One major difference in electron diffraction is the much shorter wavelength compared with X-rays, *e.g.* 0.0251 Å for 200 keV electrons. This results in a small maximum diffraction angle 2θ_max_, which in turn results in a strong correlation between the unit-cell constants and the detector distance (Clabbers *et al.*, 2018 ▸). It is difficult to calibrate the detector distance reliably: hysteresis effects in the electro-optical system of the transmission electron microscope make it difficult to return to the exact same state between the calibration powder sample and the sample in question. Any uncertainty in detector distance leads to an increased uncertainty of the unit-cell parameters. However, as the unit-cell parameters are only used to predict the spot positions, a systematic error does not render the data quality useless, and the structure can still be solved. Nevertheless, accurate prediction of the spot positions leads to better modelling of the reflection background and the reflection profile, and therefore to a more accurate estimate of both the signal and its standard deviation.

During refinement, an inaccurate cell will lead to inaccurate bond distances and bond angles. In organic and macromolecular crystallography, bond distances and bond angles typically have a high precision and can be used as restraints in order to improve model quality (Engh & Huber, 1991 ▸). This high precision can and has been used as model validation, and to determine systematic errors in the unit-cell constants, as implemented in the program *WHATCHECK* (Hooft *et al.*, 1996 ▸). The program *REFMAC5* can make use of geometric restraints to improve the unit-cell parameters during model refinement (Kovalevskiy *et al.*, 2018 ▸).

Here, we applied these same principles to electron diffraction and developed the program *CELLOPT* for small-molecule structure refinement using *SHELXL* (Sheldrick, 2015*a* ▸). *CELLOPT* reads an input file in *SHELXL* format and optimizes the unit-cell parameters on the basis of the bond distance and angular restraints. We illustrate the use of *CELLOPT* with the structures of a previously unpublished Nd^III^-based metal–organic framework (MOF) we named Vie-1 and the antiviral medication oseltamivir, for which only an X-ray structure is available. Furthermore, we tested the cell optimization routine using various examples from the literature, including several microcrystal electron diffraction (MicroED) structures of organic pharmaceutical compounds (van Genderen *et al.*, 2016 ▸; Gruene *et al.*, 2018 ▸; Jones *et al.*, 2018 ▸; Clabbers *et al.*, 2019 ▸; Bruhn *et al.*, 2021 ▸). We provide an overview of the structures used in this work in Table 1 ▸. Finally, we discuss how the results from *CELLOPT* can be combined with data processing in *XDS* (Kabsch, 2010*b* ▸) to correct for optical distortions that may potentially be present in transmission electron microscopy (Capitani *et al.*, 2006 ▸). This approach is different from previous publications that focus on treatment of elliptical distortions (Mugnaioli *et al.*, 2009 ▸; Ångström *et al.*, 2018 ▸; Clabbers *et al.*, 2017 ▸, 2018 ▸; Bücker *et al.*, 2021 ▸).

## 2. Methods

The principal idea is the following: model refinement improves the atomic coordinates by minimizing the discrepancy between the calculated and observed diffraction intensities. When geometric restraints for the structural model are present, the discrepancies between the targeted and observed bond distances and angles are added to the target function for optimization. The unit-cell parameters affect the bond distances and angles, as well as the calculated diffraction intensities. Hence, the unit-cell parameters can be modified to optimize the model geometry.

We implemented two different versions of the program *CELLOPT*: one implementation in Python and one in C++. Within this manuscript we refer to *CELLOPT(PY)* and *CELLOPT(C++)* to differentiate between these implementations. Both versions are available on github: *CELLOPT(PY)* (Luebben, 2017 ▸) at https://github.com/JLuebben/CellOpt and *CELLOPT(C++)* (Gruene, 2020 ▸) at https://github.com/tgruene/cellopt. Both versions honour the respective lattice constraints for the unit-cell parameters (Table 2 ▸), which can be relaxed to *P*1 by the user. *CELLOPT(C++)* runs within a matter of milliseconds in the cases presented in this manuscript. The output is suitable for scripting in order to combine the minimization with refinement with *SHELXL* (Sheldrick, 2015*a* ▸) of the model with the new unit-cell parameters. *CELLOPT(PY)* automates this iteration. Both programs read a *SHELX* RES file, honour grouping into residues with the RESI command, and make use of DFIX (1,2 distances) and DANG (1,3 distances) restraints.

### 2.1. *CELLOPT(PY)*: Python implementation

The Python implementation *CELLOPT(PY)* uses a multi-level hill-climbing algorithm to find the unit-cell parameters yielding the best agreement between molecular geometry and geometry restraints. The algorithm can be separated into two principle steps:

(1) Optimizing unit-cell parameters while keeping atomic coordinates constant.

(2) Optimizing atomic coordinates while keeping unit-cell parameters constant.

The geometry restraints are effectively used as the data against which the model is refined. The separation between the two steps is made to speed up the program. An alternative mode where both steps are performed simultaneously is available but not recommended because no significant improvement compared with the much quicker two-step mode was observed. By default, *CELLOPT(PY)* respects the lattice constraints but can also refine all six unit-cell parameters, *e.g.* to validate the crystal system. It can create plots to document the optimization process.

#### 2.1.1. Optimizing unit-cell parameters

Unit-cell parameters are optimized by systematically modifying each individual cell parameter and subsequently computing the weighted mean difference between the atomic coordinates and the geometry restraints. The weights of each restraint are used as the weights for the mean as well. If a modified cell yields a structure that is less discrepant compared with the geometry restraints, it is used as the new cell for subsequent iterations; otherwise it is discarded. If more than one unit-cell parameter modification yields better agreement, the modification with the largest improvement is kept and all other modifications are discarded. The process is then repeated until no more improvements are found. Which parameters are modified depends on the crystal class to ensure that the class does not change. The program provides the option to override the crystal class to quickly test different scenarios. The initial step size for modifying the bond lengths or angles is 0.1 Å or 0.1°, respectively. If each parameter is tested for the given crystal class without improving the fitting criterion, the step size is halved. The process is aborted after ten cycles without improvement.

#### 2.1.2. Optimizing atomic coordinates

After the process described in Section 2.1.1 converges, a new *SHELXL* input file including the optimized cell parameters is created and *SHELXL* is started. The resulting atomic geometry is then used as input for the next cycle of optimization of the unit-cell parameters as in Section 2.1.1. The process is repeated until it converges within numerical limits or is aborted after 25 iterations.

#### 2.1.3. Combined mode

An optional mode is provided that performs both previously described steps at once, by performing a *SHELXL* refinement step after each unit-cell parameter modification step. Instead of the agreement between geometry restraint and atomic coordinates, the improvement in *wR*2 is used to determine which unit-cell modification to keep.

### 2.2. *CELLOPT(C++)*: C++ implementation

The C++ implementation *CELLOPT(C++)* uses the Broyden–Fletcher–Goldfarb–Shanno (BFGS) algorithm implemented as BFGS2 in the GNU Scientific Library GSL (Galassi *et al.*, 2022 ▸). The BFGS algorithm is faster than the classical Newton algorithm and more robust with respect to the choice of the step sizes governing how much the unit-cell parameters are changed during the optimization process. The latter means that the user can safely rely on the default step size 0.01 (Wikipedia Contributors, 2021 ▸).

*CELLOPT(C++)* accepts the +filename syntax of *SHELXL*, by which restraints can be stored in separate files.

#### 2.2.1. Target function and gradients

*CELLOPT(C++)* modifies the unit-cell parameters in order to minimize the following target function: 

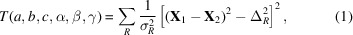

where **X**_1_ and **X**_2_ are the orthogonal coordinates of two atoms, Δ_*R*_ is the target distance for the restraint *R* between the two atoms from DFIX and DANG commands, and σ_*R*_ is the weight for the target value *R* from DFIX and DANG commands, with defaults 0.02 and 0.04, respectively.

The target function computes the difference between the observed distance between two atoms, (**X**_1_ − **X**_2_)^2^, and the desired distance of the corresponding restraint *R*. The square of the difference is weighted by the inverse variation of the restraint. This value is summed over all restraints provided in the RES file. Intuitively, one might sum 1/σ||**X**_1_ − **X**_2_| − Δ_*R*_|. The two functions have the same minimum position. However, the modulus function | · | is computationally more time consuming than the square, and the derivatives of the square are much easier to compute. The explicit forms of all derivatives are listed in Appendix *A* [App appa].

The BFGS algorithm is a gradient-based minimization algorithm, which uses the first and second derivatives in order to determine how much to modify each of the unit-cell parameters in order to move towards the minimum of the target function (Wikipedia Contributors, 2021 ▸). As common to most gradient-based minimization algorithms, only local minima can be found, and no information is available about whether this coincides with the global minimum. The general form of the gradient with respect to one of the unit-cell parameters reads 

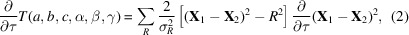

where τ stands for one of the six unit-cell parameters *a*, *b*, *c*, α, β or γ.

Crystal systems other than triclinic are implemented with their respective constraints (Massa, 1999 ▸). The user must provide one of the crystal systems (see Table 2 ▸ for constraints and command line options). These constraints simplify the above equations and reduce the number of derivatives.

The gradient is a function with the same number of dimensions as there are parameters: in the case of *CELLOPT(C++)*, up to six unit-cell parameters, depending on the crystal system. Although it is a multi-dimensional function, the gradient is a one-dimensional direction pointing down the steepest direction of the target function. The BFGS algorithm takes a step along the gradient towards the target function value. The optimal step is calculated as part of the BFGS algorithm, to avoid moving beyond the minimum position. The target function value and the gradient are computed again at the new position, and the step is repeated until the local minimum of the target function is reached within a desired small epsilon cut-off.

### 2.3. Generation of geometric restraints

The *SHELXL* command WPDB -1 generates a coordinate file in Protein Data Bank (PDB) format, including hydrogen atoms. In the demonstrations discussed here, this PDB file was converted to MOL2 format with *OpenBabel* (O’Boyle *et al.*, 2011 ▸). Geometric restraints were generated from the MOL2 file with the *GRADE* server (Global Phasing, 2017 ▸). The MOL2 format ensures consistent atom names between the input file and the restraints.

### 2.4. Iterative cell optimization

**Figure 1:**
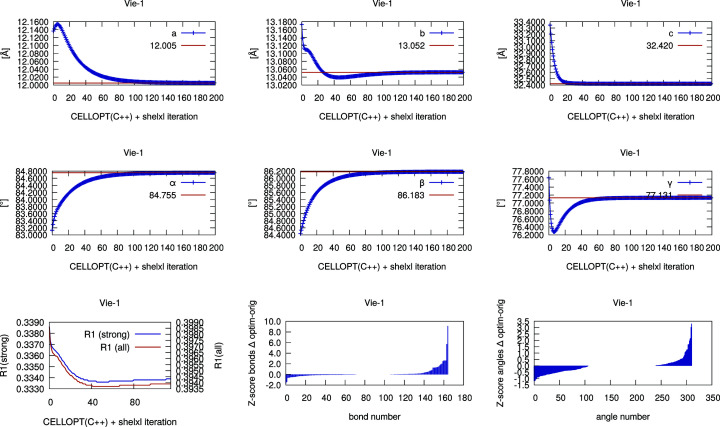
Iterative cell optimization of Vie-1, an Nd^III^-based metal–organic framework (triclinic space group 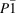 ): *a*, *b*, *c* axes, α, β, γ angles, *R*1 values, and *Z* scores. *Z* scores show the difference of the models after and before cell optimization with *CELLOPT(C++)*. Negative values refer to a lower *Z* score after optimization and thus to an improvement of the geometry.

**Figure 2:**
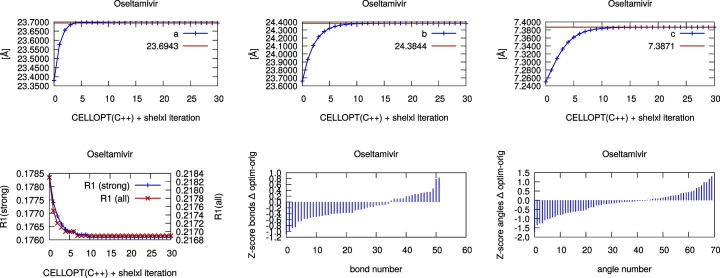
Iterative cell optimization of oseltamivir (orthorhombic space group *P*2_1_2_1_2_1_): *a*, *b*, *c* axes, *R*1 values, and *Z* scores.

Iterative cell optimization is built into the Python version of *CELLOPT*. The C++ implementation can be used for iterative optimization with a shell script that alternates between *CELLOPT* and a run of *SHELXL* with the new cell. We extracted the unit-cell parameters and the *R*1 values (strong and all reflections) for each iteration. The plots in the supporting information and Figs. 1 and 2 below were generated from these data for each iteration. The *Z* scores for bonds and angles (Joosten *et al.*, 2014 ▸) were generated with the program *MOGUL* (Bruno *et al.*, 2004 ▸). The scores were sorted in descending order, so that identical points on the chart may not correspond to the same bond or angle in each structure. An example BASH script is provided in the supporting information Section 2.

### 2.5. Cell optimization with *REFMAC5*

Table 3 ▸ compares the results of *CELLOPT(PY)* and *CELLOPT(C++)* with the original unit-cell parameters. It also includes the results from cell optimization with *REFMAC5* (CCP4 7.1.014: *REFMAC* version 5.8.0267; Kovalevskiy *et al.*, 2018 ▸). Note that lattice refinement in *REFMAC5* is meant for validation, not for determination of the unit-cell parameters. The PDB file was created with the *SHELXL* command WPDB -1 and curated with *PDBSET* (CCP4; https://www.ccp4.ac.uk/html/pdbset.html). *PDBSET* was used to set a chain ID. The same restraints as generated by *GRADE* (Global Phasing, 2017 ▸) were provided to *REFMAC5* with the command line option LIB_IN grade-dict.cif, where grade-dict.cif is the filename of the mmCIF file generated by the *GRADE* server. In the case of several moieties, the CIF dictionaries were concatenated into a single one. A minimum script file reads 
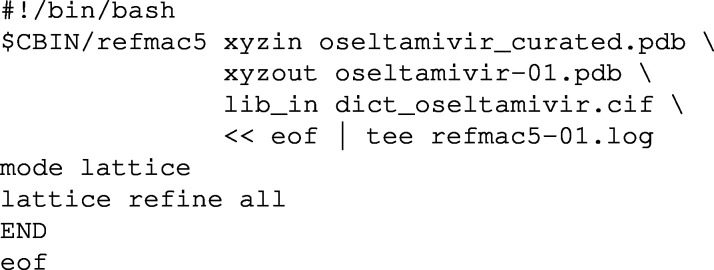


Table 3 ▸ also provides the respective runtimes. For *CELLOPT(C++)*, a single run is a matter of milliseconds on an AMD Ryzen 5 or INTEL Core i7. The numbers of iterations with *SHELXL* are given in brackets.

### 2.6. Comparison of results, *Z*-score plots

The plots in Figs. 1 ▸, 2 ▸ and S1–S10 show the development of the unit-cell parameters during the iterative cell optimization between *CELLOPT(C++)* and *SHELXL*, as well as the value of *R*1 after each iteration. They also show the *Z* scores of the bonds and angles. *Z* scores were generated with *MOGUL* (Bruno *et al.*, 2014 ▸) with automated assignment of bond types and angle types. A low *Z* score indicates a good match with the average bonds and angles. The plots show the difference of the respective *Z* scores before and after cell optimization, so that a negative value denotes an improvement of the geometry. Note that some misassignments may occur. For example, the high positive values for the bonds of Vie-1 relate to the different classification of the C—O distances in the conjugate carboxyl groups.

### 2.7. Synthesis of MOF Vie-1

All experiments were performed in air and solvents were used as received. Nd(NO_3_)_3_·6H_2_O was purchased from Sigma Aldrich and 4,4′,4′′,4′′′-(pyrene-1,3,6,8-tetrayl)tetrabenzoic acid was synthesized according to a literature procedure (Wang *et al.*, 2016 ▸).

*Synthesis*. In a Teflon-lined hydrothermal reactor, Nd(NO_3_)_3_·6H_2_O (32 mg, 1 equiv.) and 4,4′,4′′,4′′′-(pyrene-1,3,6,8-tetrayl)tetrabenzoic acid (25 mg, 0.5 equiv.) were dissolved in 10 ml of dimethyl formamide:dioxane:H_2_O (2:1:1) and heated over a period of 14 h to 353 K. The reaction mixture was then kept at 353 K for 24 h and subsequently cooled to 293 K over a period of 14 h, at which point crystals suitable for ED were obtained.

### 2.8. Oseltamivir

Dry powder of oseltamivir was kindly provided by Roche. A grain of the powder was deposited on a glass cover slide and dispersed with a fine brush. A Cu grid with lacey carbon (200 mesh, 2.3 mm diameter; FIAS, Austria) was dropped onto the powder. A second glass cover slide was placed on top and pressure was applied with a finger. Data were collected at *T* = 184 K from seven crystals, at an effective detector distance of 404 mm. Data from three different crystals were merged with *XSCALE* (Kabsch, 2010*a* ▸) for structure solution and refinement to increase data completeness. The structure was solved with *SHELXT* and refined with *SHELXLE*/*SHELXL* (Kabsch, 2010*b* ▸; Hübschle *et al.*, 2011 ▸; Sheldrick, 2015*a* ▸,*b* ▸). The X-ray structure determined at Roche was used for comparison of the hydrogen-bonding network.

### 2.9. Data collection and processing

A lacey carbon grid (Ted Pella) was scraped over the wall of a 14 ml plastic tube containing the crystals. Data were collected with a Phillips CM200 equipped with a 1024 × 512 pixel JUNGFRAU detector (Fröjdh *et al.*, 2020 ▸). Data from three different crystals of the MOF Vie-1 were merged to increase data completeness.

### 2.10. CSD codes and raw data

The new models based on the optimized geometry were deposited at the Cambridge Structural Database (CSD; Groom *et al.*, 2016 ▸) with CSD codes 2124898 for Vie-1 and 2124897 for Oseltamivir. Raw data in CBF format, including XDS input files to repeat processing and scaling, are available at https://doi.org/10.5281/zenodo.5734130.

## 3. Results

**Figure 3:**
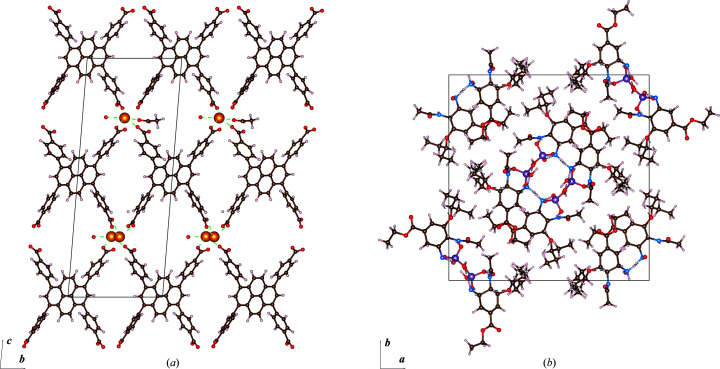
Structural models of Vie-1 and oseltamivir after cell optimization and refinement. (*a*) Structure of Vie-1 shown in the crystallographic *bc* plane, illustrating the framework formed through electrostatic interactions coordinating the Nd metal ions with the organic linkers (C_40_O_8_). Restraints on bond lengths and angles were generated for (pyrene-1,3,6,8-tetrayl)tetrabenzoic acid as described in Section 2.3. (*b*) Structure of the organic pharmaceutical oseltamivir (C_16_N_2_O_4_) shown in the *ab* plane, where the crystal packing is formed by hydrogen-bonding interactions between oseltamivir and (PO_4_)^−^. Idealized restraints were generated for bond lengths and angles of the oseltamivir compound. Colour coding for the different atoms is white, brown, blue, red, purple and orange for hydrogen, carbon, nitrogen, oxygen, phosphor and neodymium, respectively. Figures were made using *VESTA* (Momma & Izumi, 2011 ▸).

Unit-cell parameters determined from electron diffraction data are typically one order of magnitude less precise than those from X-ray diffraction (Mugnaioli *et al.*, 2009 ▸; Ångström *et al.*, 2018 ▸). We introduced an iterative cell optimization and refinement procedure, based on minimizing the deviation from idealized geometric restraints for 1,2 and 1,3 bond distances. We tested our program using electron diffraction data of an organic pharmaceutical compound and a MOF-type material (Fig. 3 ▸). Furthermore, we tested our program against several previously solved structures from the CSD (van Genderen *et al.*, 2016 ▸; Gruene *et al.*, 2018 ▸; Jones *et al.*, 2018 ▸; Clabbers *et al.*, 2019 ▸; Bruhn *et al.*, 2021 ▸). We summarize our results in Table 1 ▸. The table lists the *R*1 factors and Δ*R*_complete_ = *R*_complete_ − *R*1 of the original model compared with the optimized model. *R*_complete_ is more sensitive to chemically meaningful changes in a structure and is a measure of overfitting against errors in the data (Luebben & Gruene, 2015 ▸). In all cases, except for the methylene blue derivative, *R*1 shows a slight decrease after cell optimization and the data show no considerable sign of overfitting from introducing the geometrical restraints,

Furthermore, we illustrate the change of cell parameters, *R*1 value and the *Z* scores for 1,2 and 1,3 bond distances for each iteration of cell optimization in *CELLOPT* and refinement using the new cell in *SHELXL*. We show the results for oseltamivir (Fig. 2 ▸) and the metal–organic framework Vie-1 (Fig. 1 ▸) as examples. Subsequent plots for all literature structures are presented in the supplementary Figs. S1–S10. *Z* scores are considered better quality indicators than, for example, *R*1 values (Joosten *et al.*, 2014 ▸; Tickle, 2007 ▸). In all cases, the unit-cell parameters converge to stable values within about 20 iterations and show an improvement in *Z* scores for bond lengths and angles.

## 4. Discussion

Organic structures usually have highly conserved bond distances with very small deviations (Engh & Huber, 1991 ▸). This information can be used to improve the accuracy of unit-cell parameters in crystal structures determined from electron diffraction data. Such data typically have low precision and low accuracy in unit-cell parameters, compared with structures determined from X-ray diffraction data. Inorganic structures have a tendency to display higher variability in bond distances and bond angles. In inorganic chemistry, electron diffraction data can be complemented by more precise lattice parameters from powder X-ray diffraction (McCusker & Baerlocher, 2013 ▸). Our work presents two implementations of an optimization algorithm to improve the accuracy of the unit-cell parameters based on idealized geometrical restraints, independent of additional experimental characterization of the lattice parameters. We show a gradual change in unit-cell parameters approaching convergence using the cell optimization routine, and a slight improvement of the model *R* factors whilst not overfitting the data (Table 1 ▸, Fig. 1 ▸, Fig. 2 ▸ and Figs. S1–S10). We optimized the unit-cell parameters for a novel Vie-1 MOF, the pharmaceutical compound oseltamivir, and several previously determined structures (van Genderen *et al.*, 2016 ▸; Gruene *et al.*, 2018 ▸; Jones *et al.*, 2018 ▸; Clabbers *et al.*, 2019 ▸; Bruhn *et al.*, 2021 ▸). Our approach is not limited to MicroED data (Nannenga *et al.*, 2014 ▸), but may also be applied in structure refinement using related 3D ED techniques (Dorset, 1995 ▸; Kolb *et al.*, 2007 ▸; Zhang *et al.*, 2010 ▸) or serial electron diffraction data (Smeets *et al.*, 2018 ▸; Bücker *et al.*, 2021 ▸).

Discrepancies between observed and predicted spot positions can be mapped onto the detector surface and can be used to correct for distortions of the detector from ideality. This used to be common practice for wireframe detectors, for the glass fibre optics in CCD detectors, and for modular detectors used at some beamlines and free-electron lasers (Parkhurst *et al.*, 2014 ▸; Wagner *et al.*, 2016 ▸; Ginn & Stuart, 2017 ▸; Brewster *et al.*, 2018 ▸). In transmission electron microscopy, such distortions can originate from the lens system of the microscope (Capitani *et al.*, 2006 ▸) and can, for example, cause elliptical distortions (Mugnaioli *et al.*, 2009 ▸; Ångström *et al.*, 2018 ▸; Clabbers *et al.*, 2017 ▸, 2018 ▸; Bücker *et al.*, 2021 ▸). Taking the shifts into account should result in better background estimates and a better *I*/σ_*I*_. We did attempt to show this with our data. However, the ellipticity of our instrument (*A*/*B* − 1 for the major and minor axes of the ellipse from an Al-powder pattern) varies between 0.0005 and 0.003. This is too little for a significant improvement on the data. Because the problem has been pointed out at workshops and discussions, we provide a work-flow based on the program *XDS* in the supporting information. This approach is independent of the type of distortion, as long as they do not produce overlaps of the distorted pixels (one-to-one distortion). Originally, we did observe a significant improvement in data quality for oseltamivir. However, when we reprocessed the data with the cell from *CELLOPT*, it turned out that the detector distance was set to 432 mm instead of 406 mm in the *XDS* input script. Correcting this error made the difference become insignificant. However, *CELLOPT* was helpful in spotting a user-induced systematic error, rather than an instrumental systematic error.

## 5. Conclusions

The cell optimization routine benefits the refinement of small-molecule structures against electron diffraction data. There are, however, some drawbacks that can limit the usefulness of this routine. As already mentioned above, inorganic structures can show higher variance and more disorder, which makes a routine based on geometrical restraints less effective or even inappropriate. In a similar way, the crystal packing and 3D geometry of the molecule of interest dictate how well defined the restraints are in each direction and how this affects the resulting lattice parameters. For example, a relatively flat molecule that is only well ‘restrained’ in two dimensions would be lacking along the third crystallographic direction, depending on the crystal packing. Incomplete data with a missing wedge of reflections, which is not uncommon for electron diffraction, can increase the uncertainty in unit-cell parameters along the crystallographic direction with the missing information. The cell optimization routine can work well for such incomplete cases. For example, despite the paracetamol structure only having 35% completeness (Gruene *et al.*, 2018 ▸), it rapidly converges and shows improved model *R* factors and *Z* scores after optimization (Fig. S3). Both crystallographic and chemical understanding of the individual system under consideration are required in order to decide whether cell optimization will improve the accuracy of the unit-cell parameters.

## 6. Related literature

The following additional references are cited in the supporting information: Evans (2006 ▸), Evans & Murshudov (2013 ▸) and Winn *et al.* (2011 ▸).

## Supplementary Material

Supplemental Information, plots for all structures, data and model statistics for Vie-1 and Oseltamivir. DOI: 10.1107/S160057672200276X/yr5082sup1.pdf

Crystal structure: contains datablock(s) MOF_Vie-1, oseltamivir--opt. DOI: 10.1107/S160057672200276X/yr5082sup2.cif

Structure factors: contains datablock(s) MOF_Vie-1. DOI: 10.1107/S160057672200276X/yr5082MOF_Vie-1sup3.hkl

Structure factors: contains datablock(s) oseltamivir--opt. DOI: 10.1107/S160057672200276X/yr5082oseltamivir--optsup4.hkl

CBF files for all diffraction data sets included. The repository also includes XDS files for processing and XSCALE files for data scaling.: https://doi.org/10.5281/zenodo.5734130

CCDC references: 2124897, 2124898

## Figures and Tables

**Table 1:** List of structures used in this study, together with the *R*1 values with the original cell and the optimized cell *R*1 value refers to all data; *R*1 in brackets (second line) refers to strong data with *I*/σ_*I*_ ≥ 2. MBD: methylene blue derivative; LSPD: (+)-limaspermidine. Δ*R*_complete_ = *R*_complete_ − *R*1.

		Original		Optimized	
CSD refcode	Name	*R*1	Δ*R*_complete_	*R*1	Δ*R*_complete_
N/a	Vie-1	39.742	1.260	39.231	1.309
		(34.524)	(1.418)	(34.069)	(1.478)
N/a	Oseltamivir	22.024	2.132	21.816	2.176
		(19.115)	(2.158)	(18.832)	(2.203)
PROGST16	Progesterone	16.18	2.409	16.14	2.39
		(13.25)	(2.407)	(13.23)	(2.439)
LIMZAL01	MBD	30.88	2.506	31.95	2.129
		(27.28)	(3.192)	(28.77)	(2.282)
COTZAN07	Paracetamol	29.87	1.944	28.48	1.496
		(27.79)	(2.113)	(26.00)	(2.240)
CBMZPN28	Carbamazepine	28.659	3.400	28.611	3.473
		(26.485)	(3.325)	(26.414)	(3.395)
BISGAO	Epicorazine A	18.662	2.312	18.636	2.323
		(17.523)	(2.334)	(17.49)	(2.345)
IRELOH01	IRELOH	17.008	2.642	16.975	2.617
		(15.783)	(2.588)	(15.715)	(2.557)
CINCHO11	Cinchonine	21.231	1.994	21.109	1.983
		(21.210)	(1.979)	(21.080)	(1.968)
CAHKUU01	LSPD	25.255	1.174	25.097	1.192
		(20.962)	(1.291)	(20.768)	(1.309)

**Table 2:** Constraints for crystal systems and command line options for the C++ implementation of *CELLOPT* (Massa, 1999 ▸) ‘c.l. option’ denotes the command line option to set the respective crystal system. ‘#var.’ denotes the number of independent variables for the optimization algorithm [*cf.* equation (1)].

Crystal system	Constraints	c.l. option	#var.
Triclinic	None	-xa	6
Monoclinic	α = γ = 90°	-xm	3
Orthorhombic	α = β = γ = 90°	-xo	3
Hexagonal	*a* = *b*, α = β = 90°, γ = 120°	-xh	2
Tetragonal	*a* = *b*, α = β = γ = 90°	-xt	2
Cubic	*a* = *b* = *c*, α = β = γ = 90°	-xc	1

**Table 3:** Comparison of optimized cell parameters for *CELLOPT(PY)*, *CELLOPT(C++)* and *REFMAC5* *t*: runtime for the optimization of the specific program. For *CELLOPT(C++)*, the number of iterations between *CELLOPT(C++)* and *SHELXL* refinement is given in brackets.

Molecule	*a* (Å)	*b* (Å)	*c* (Å)	α (°)	β (°)	γ (°)	*t* (s)
Vie-1							
Original	12.136	13.173	33.346	83.130	84.435	77.633	–
*CELLOPT(PY)*	12.096	13.030	32.418	84.033	85.426	76.932	83
*CELLOPT(C++)*	12.005	13.052	32.420	84.755	86.183	77.131	250 (199)
*REFMAC5*	12.136	13.173	33.346	83.130	84.440	77.630	15

Oseltamivir							
Original	23.380	23.660	7.250	90	90	90	–
*CELLOPT(PY)*	23.683	24.344	7.297	90	90	90	18
*CELLOPT(C++)*	23.694	24.384	7.387	90	90	90	20 (30)
*REFMAC5*	23.465	23.910	7.265	90	90	90	16

Progesterone							
Original	10.277	12.555	13.504	90	90	90	–
*CELLOPT(PY)*	10.264	12.576	13.569	90	90	90	16
*CELLOPT(C++)*	10.206	12.5423	13.561	90	90	90	9 (15)
*REFMAC5*	10.033	12.573	13.947	90	90	90	11

MBD							
Original	40.070	16.565	13.753	90	98.543	90	–
*CELLOPT(PY)*	40.067	16.486	14.433	90	101.786	90	168
*CELLOPT(C++)*	40.208	16.650	14.789	90	103.272	90	14 (14)
*REFMAC5*	40.070	16.565	13.753	90	98.543	90	15

Paracetamol							
Original	6.9620	9.1768	11.5564	90	98.8212	90	–
*CELLOPT(PY)*	7.224	9.855	11.113	90	101.821	90	8
*CELLOPT(C++)*	7.226	8.561	12.073	90	100.871	90	26 (55)
*REFMAC5*	6.962	9.177	11.556	90	98.820	90	14

Carbamazepine							
Original	7.578	11.176	13.991	90	93.077	90	–
*CELLOPT(PY)*	7.525	10.964	13.854	90	92.508	90	20
*CELLOPT(C++)*	7.571	10.955	13.932	90	92.623	90	46 (65)
*REFMAC5*	7.578	11.176	13.991	90	93.080	90	14

Epicorazine A							
Original	10.996	12.452	13.218	90	90	90	–
CELLOPT(PY)	11.849	12.733	13.071	90	90	90	11
CELLOPT(C++)	10.90014	12.73995	13.0874	90	90	90	6 (20)
REFMAC5	10.997	12.581	13.187	90	90	90	14

IRELOH							
Original	8.015	10.015	17.703	90	90	90	–
*CELLOPT(PY)*	7.9994	9.9555	18.0188	90	90	90	8
*CELLOPT(C++)*	8.016	10.029	17.652	90	90	90	1 (5)
*REFMAC5*	8.010	10.063	17.638	90	90	90	14

Cinchonine							
Original	10.710	7.060	11.150	90	109.665	90	–
*CELLOPT(PY)*	10.666	7.069	11.147	90	109.318	90	17
*CELLOPT(C++)*	10.647	7.084	11.088	90	110.088	90	12 (20)
*REFMAC5*	10.710	7.060	11.150	90	109.660	90	15

LSPD							
Original	7.620	13.880	15.200	90	90	90	–
*CELLOPT(PY)*	7.573	13.755	15.062	90	90	90	21
*CELLOPT(C++)*	7.598	13.753	14.934	90	90	90	14 (20)
*REFMAC5*	7.591	13.867	15.078	90	90	90	14

## Appendix A

The target function [equation (1)] of *CELLOPT(C++)* depends on the unit-cell parameters through the calculation of the orthogonal coordinates **X**_1_, **X**_2_ from their fractional coordinates *x*_1_, *y*_1_, *z*_1_ and *x*_2_, *y*_2_, *z*_2_:

The partial derivatives of equation (3) with respect to *a*, *b*, *c*, α, β and γ are as follows:
