# Supplementary material for: CELLOPT: improved unit-cell parameters for electron diffraction data of small-molecule crystals
Source: J Appl Crystallogr. 2022 Apr 29;55(Pt 3):647–55. doi: 10.1107/S160057672200276X (PMC9172032; doi:10.1107/S160057672200276X)
Supplement: Supplementary file 1 [file j-55-00647-sup1.pdf]

Supplemental Information — CELLOPT: improved unit cell parameters for electron diffraction data of small molecule crystals.

Tim Gruene,[a]* Max T.B. Clabbers,[b,§]* Jens Luebben,[c] Jia Min Chin,[d] Michael R. Reithofer,[a] Frank Stowasser[e] and André M. Alker[e]

[a] *Institute of Inorganic Chemistry, Faculty of Chemistry, University of Vienna, Austria,* [b] *Department of Materials and Environmental Chemistry, Stockholm University, Sweden,* [c] *Bruker AXS, Germany,* [d] *Institute of Physical Chemistry, Faculty of Chemistry, University of Vienna, Austria,* [§] *Present address: Department of Biological Chemistry, University of California Los Angeles, USA , and* [e] *Roche Pharma Research and Early Development, Basel, Switzerland.*

E-mail: tim.gruene@univie.ac.at, clabbers@ucla.edu

1. Data and Model Statistics

1.1. Vie-1

ED data merged from three different crystals. Data from all three crystals are detector distance 279mm. In addition, low resolution data collected from one of the crystals at 725mm detector distance. This is a new structure, and no X-ray data are available for this compound. standard uncertainties in brackets calculated as weighted mean from the cell parameters and their standard uncertainties listed in CORRECT.LP.

Table S1. *Unit cell parameters for the metal organic framework structure Vie-1 before (pre-) and after (post-) cell optimisation with CELLOPT(C++)*

	a	b	c	α	β	γ
ED pre-	12.14(15)	13.17(16)	33.35(59)	83.13(37)	84.44(55)	77.63(11)
ED post-	12.07	13.07	32.34	84.60	85.95	76.88

Table S2. *Data statistics for Vie-1 for pre-optimised data calculated with POINTLESS and AIMLESS (Winn* et al.*, 2011; Evans, 2006; Evans & Murshudov, 2013).*

**Data**	Overall	InnerShell	OuterShell
Low res. limit	12.79	2.79	0.84
High res. limit	0.83	4.55	0.83
Rmeas	0.490	0.703	1.359
Total number of observations	53778	390	1993
Total number unique	12278	76	561
$< I/\sigma(I) >$	2.7	6.2	0.9
CC(1/2)	0.532	0.469	0.792
Completeness	65.0	65.5	62.6
Multiplicity	4.4	5.1	3.6

Table S3. *Model statistics for Vie-1 pre- and post-optimisation of the unit cell parameters.*

	#data	R1	wR2	GooF	rGooF	param's	restr's
pre	12,278 (5,961)	39.82 (33.79)	66.26	1.73	1.71	456	559
post	12,278 (5,961)	39.37 (33.37)	65.72	1.71	1.69	456	559

1.2. Oseltamivir

ED data merged from three different crystals; one data set was collected in two parts after correction for drift of the crystal out of the beam. For ED pre-optim$^{\underline{n}}$: standard uncertainties in brackets calculated as weighted mean from the cell parameters and their standard uncertainties listed in CORRECT.LP. Spacegroup $P2_12_12$, i.e. all angles 90°, omitted in table.

Table S4. *Unit cell parameters for Oseltamivir before (pre-) and after (post-) cell optimisation with CELLOPT(C++), in comparison of the X-ray structure (SXRD).*

	a	b	c
SXRD	23.800(5)	24.390(5)	7.3900(15)
ED pre-optim$^{\underline{n}}$	23.41(24)	23.54(10)	7.21(4)
ED post-optim$^{\underline{n}}$	23.72	24.36	7.39

Table S5. *Data statistics for pre-optimised data calculated with POINTLESS and AIMLESS (Winn* et al.*, 2011; Evans, 2006; Evans & Murshudov, 2013).*

	Overall	InnerShell	OuterShell
Low res. limit	23.38	23.38	0.93
High res. limit	0.90	3.61	0.90
R_{meas}	0.228	0.132	0.800
Total number of observations	29086	364	2381
Total number unique	3202	66	269
$< I/\sigma(I) >$	6.7	11.3	2.5
CC(1/2)	0.992	0.993	0.362
Completeness	98.2	95.9	96.4
Multiplicity	9.1	5.5	8.9

Table S6. *Model statistics for Oseltamivir pre- and post-optimisation of the unit cell parameters.*

	#data	R1	wR2	GooF	rGooF	param's	restr's
pre	5,579 (4,190)	21.83 (17.84)	44.89	1.53	1.54	230	120
post	5,579 (4,190)	21.68 (17.62)	44.46	1.52	1.51	229	120

2. BASH script for iterative cell improvement for CELLOPT(C++)

```
#/bin/bash

if (( $# != 1)); then
        echo "res-file missing"
        echo "Usage: cellopt.sh myfile.res"
        echo " make sure, the crystal system is correct (option '-x')"
        exit
fi

# path to cellopt binary, if not in PATH
CELLOPT=~/NetBeansProjects/cellopt/dist/Release/CLang-Linux/cellopt

# first argument: res-file to work on.
myres=$1

[ -e ${myres} ] || (echo "File ${myres} does not exist, exiting" && exit )

rm mycellopt.log
rm r1.data
rm cells.txt

/bin/echo -n "00 " > r1.data
grep "^REM R1 =" ${myres} | cut -c10-16,43-48 >> r1.data
grep "^CELL " ${myres} |  cut -d'L' -f3 | tee -a cells.txt

for i in $(seq -w 99); do
# adjust the '-x' option for the crystal system
readarray -t NCELL < <(${CELLOPT} -f ${myres} -v0 -xo)
# move old cell out of the way
sed -i "s/^CELL \(.*\)/REM CELL \1\n${NCELL[0]}/" ${myres}
mv ${myres} ${myres%res}ins
shelxl ${myres%.res}
echo "Run $i: ${NCELL[1]}" >> mycellopt.log
echo "Run $i: ${NCELL[0]}" >> mycellopt.log
/bin/echo -ne "Run $i: " >> mycellopt.log
grep "REM R1 = " ${myres}  >> mycellopt.log
done

# the 'cuts' should be fine for 10-99 cycles, need adjustment when > 99 cycles
grep "R1 =" mycellopt.log | cut -c4-6,17-24,51-57 | tee -a r1.data
grep CELL mycellopt.log| cut -d'L' -f3 | tee -a cells.txt
```

3. Plots for all structures

Iterative cycling between cellopt and SHELXL leads to a conversion of the unit cell parameters, and consequently of the R1 value. The following graphs shows the development of these figures for all structures listed in Table 2.

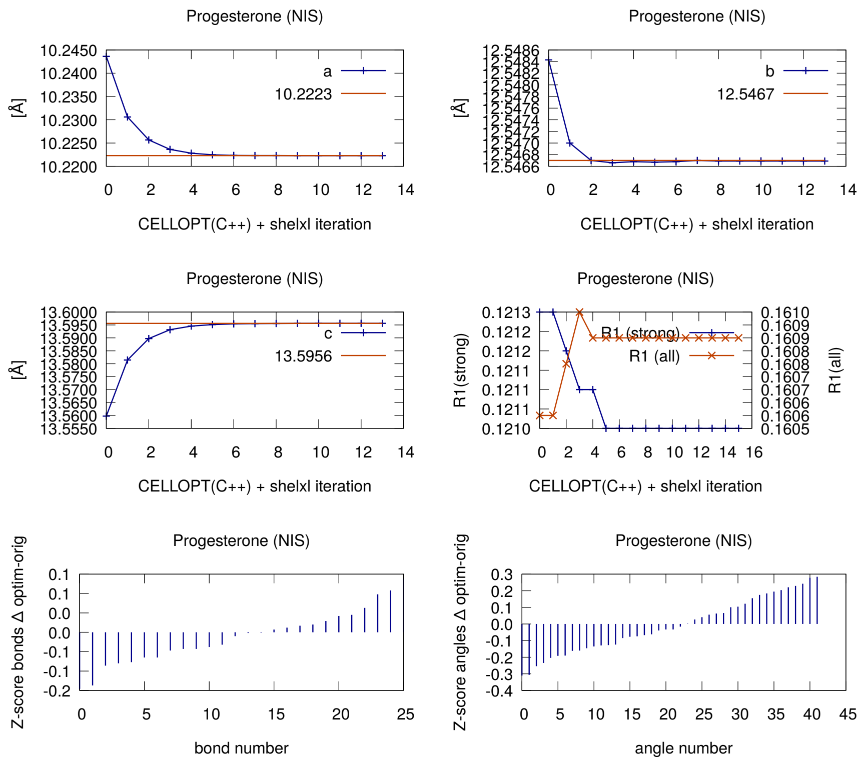

Fig. S1. Iterative cell optimisation of progesterone. Orthorhombic space group: a, b, c-axes, R1 values. Z-scores show difference of the models after and before cell optimisation with CELLOPT(C++). Hence, negative values refer to a lower Z-score after optimisation and thus to an improvement of the geometry.

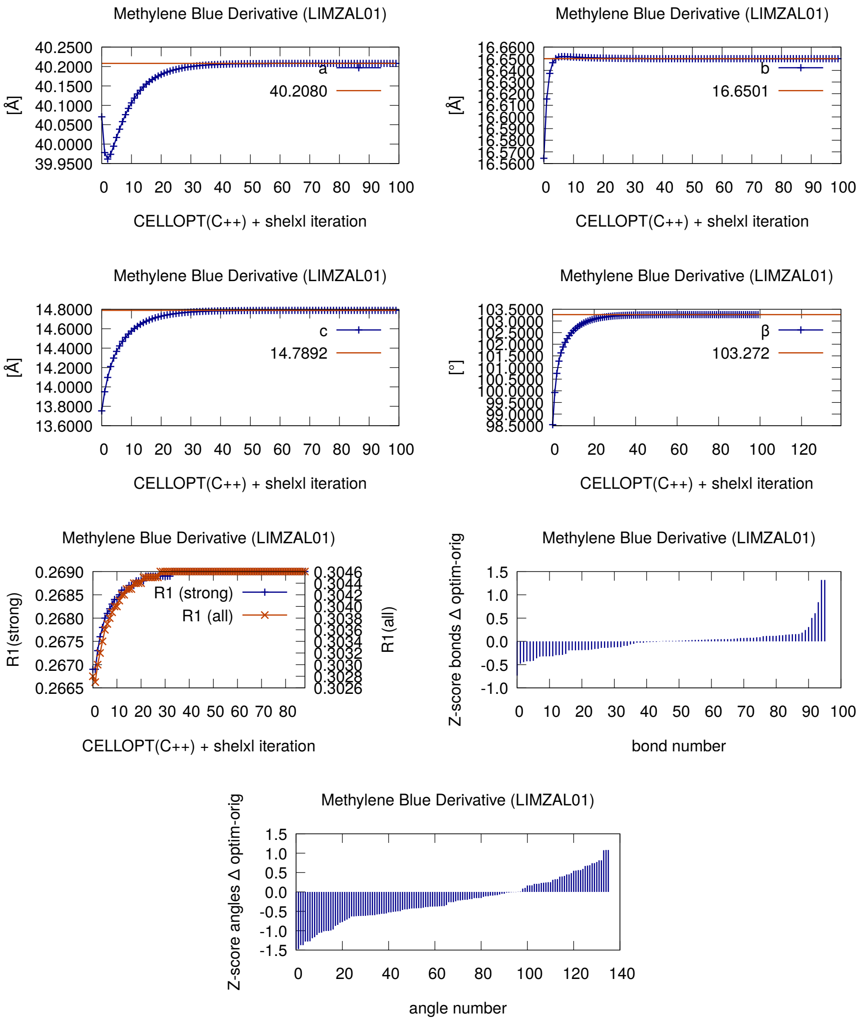

Fig. S2. Iterative cell optimisation of methylene blue derivative. Monoclinic space group: a, b, c-axes, β angle, R1 values. Z-scores show difference of the models after and before cell optimisation with CELLOPT(C++). Hence, negative values refer to a lower Z-score after optimisation and thus to an improvement of the geometry.

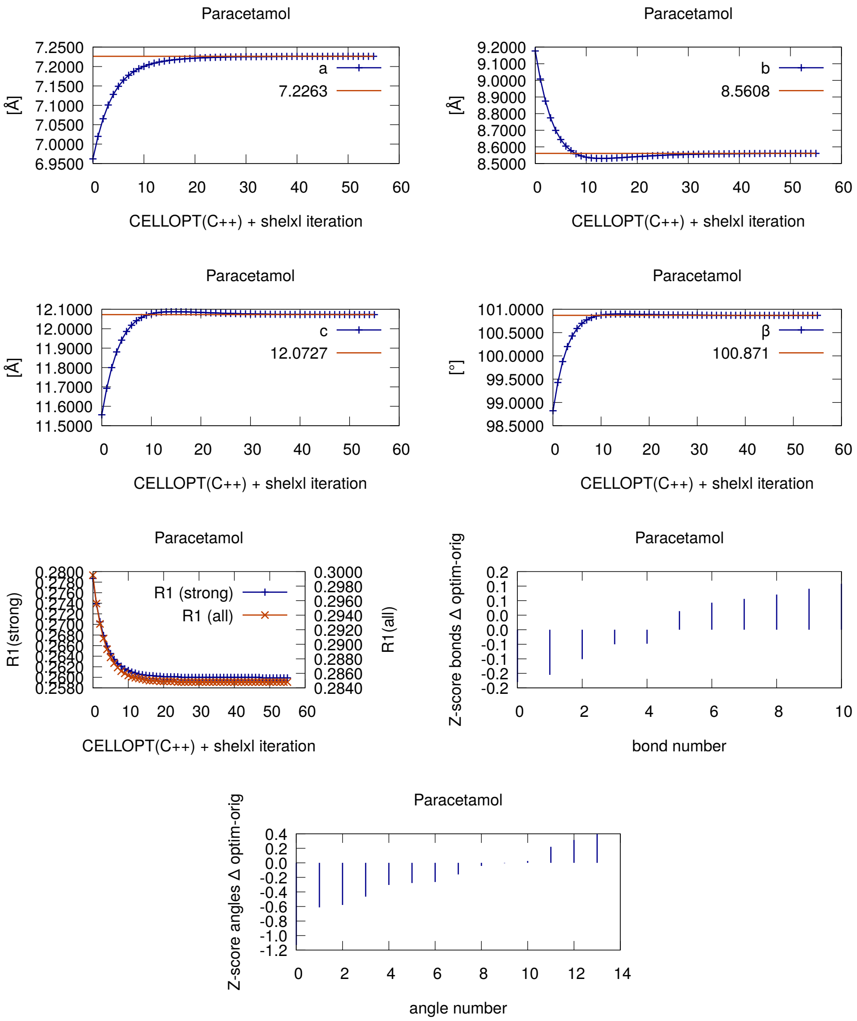

Fig. S3. Iterative cell optimisation of paracetamol. Monoclinic space group: a, b, c-axes, β angle, R1 values. Z-scores show difference of the models after and before cell optimisation with CELLOPT(C++). Hence, negative values refer to a lower Z-score after optimisation and thus to an improvement of the geometry.

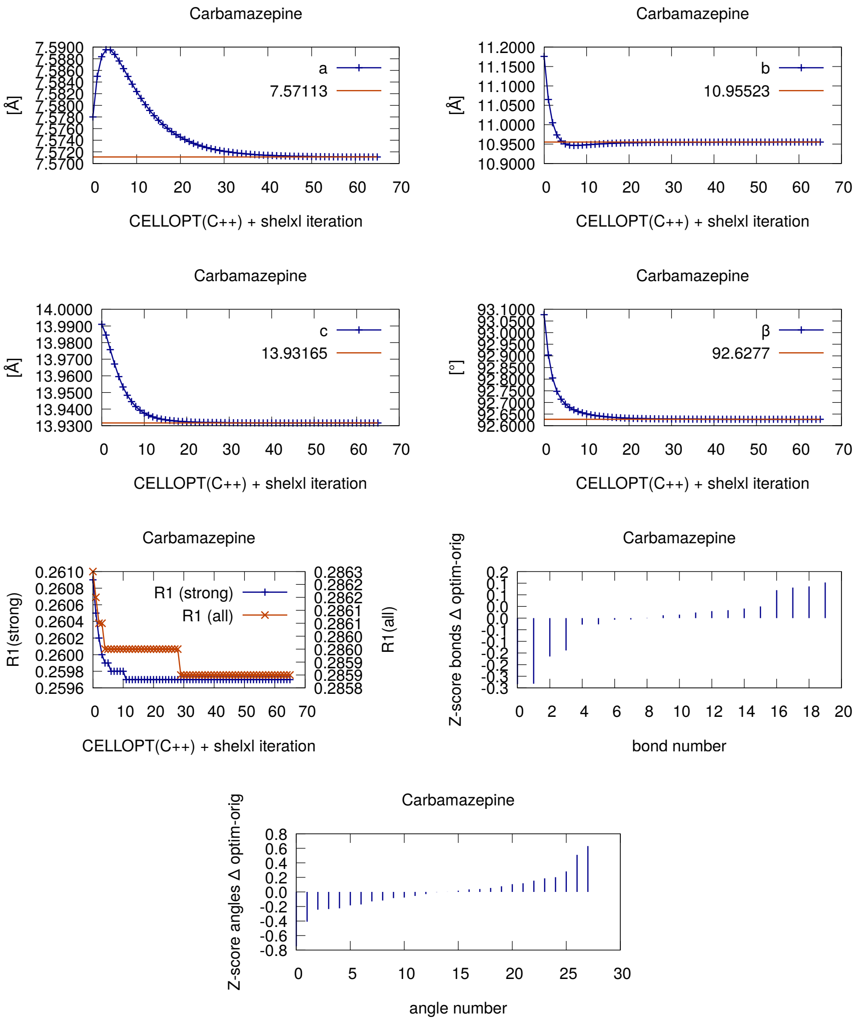

Fig. S4. Iterative cell optimisation of carbamacepine. Monoclinic space group: a, b, c-axes, β angle, R1 values. Z-scores show difference of the models after and before cell optimisation with CELLOPT(C++). Hence, negative values refer to a lower Z-score after optimisation and thus to an improvement of the geometry.

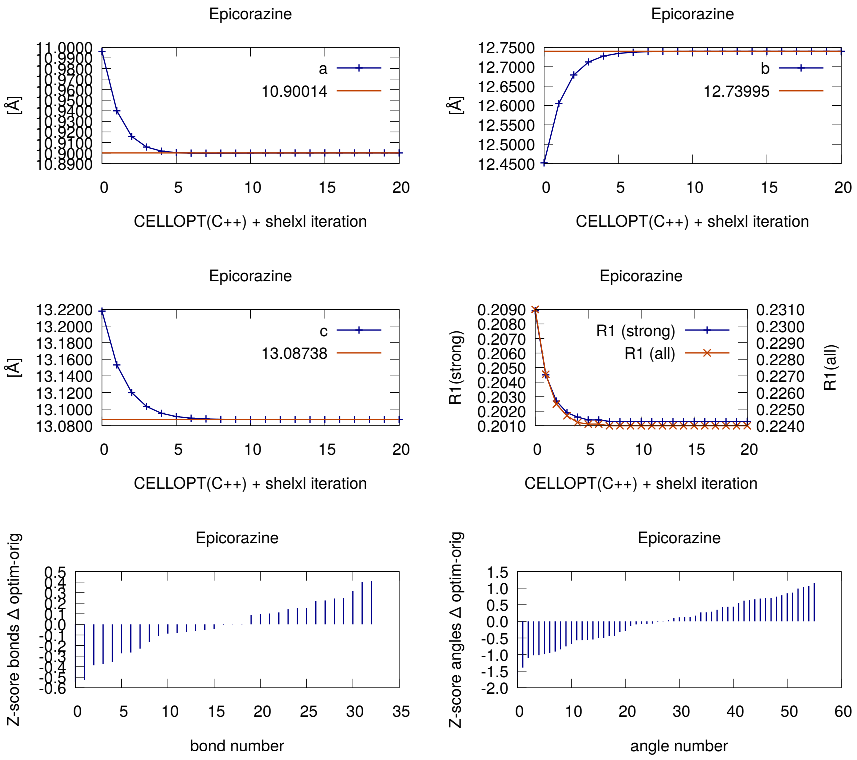

Fig. S5. Iterative cell optimisation of epicorazine A. Orthorhombic space group: a, b, c-axes, R1 values. Z-scores show difference of the models after and before cell optimisation with CELLOPT(C++). Hence, negative values refer to a lower Z-score after optimisation and thus to an improvement of the geometry.

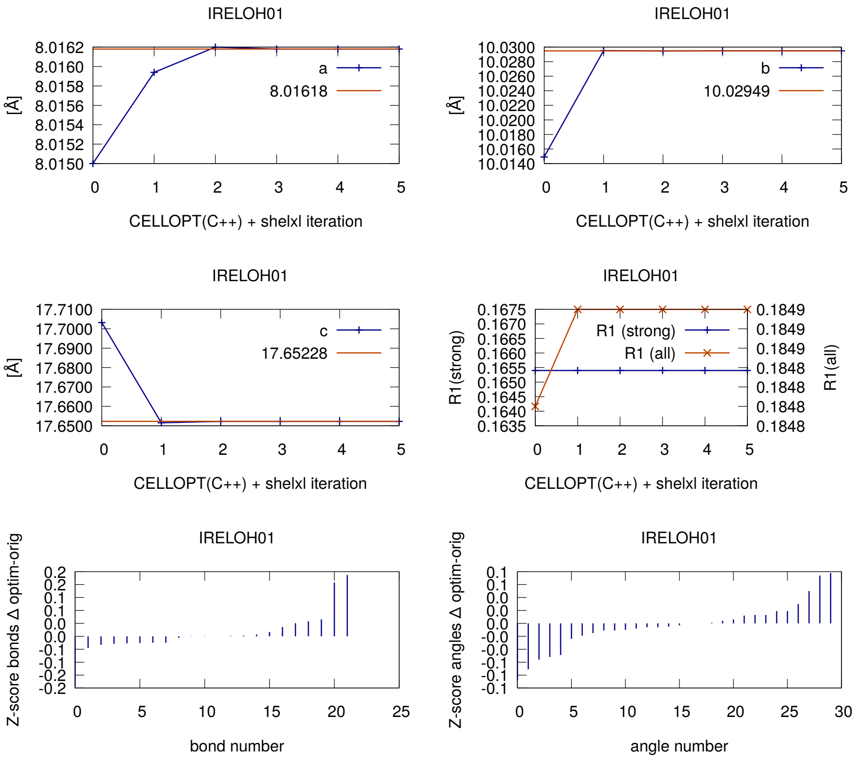

Fig. S6. Iterative cell optimisation of IRELOH. Orthorhombic space group: a, b, c-axes, R1 values. Z-scores show difference of the models after and before cell optimisation with CELLOPT(C++). Hence, negative values refer to a lower Z-score after optimisation and thus to an improvement of the geometry.

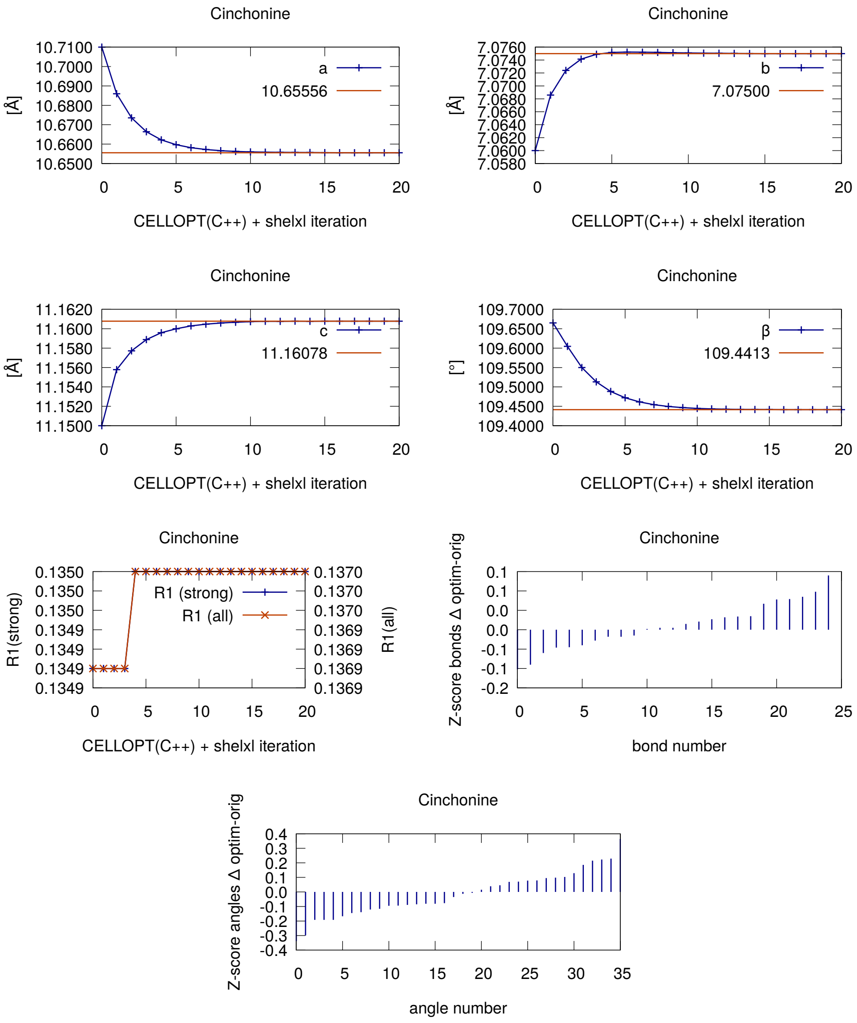

Fig. S7. Iterative cell optimisation of Cinchonine. Monoclinic space group: a, b, c-axes, β-angle, R1 values. Z-scores show difference of the models after and before cell optimisation with CELLOPT(C++). Hence, negative values refer to a lower Z-score after optimisation and thus to an improvement of the geometry.

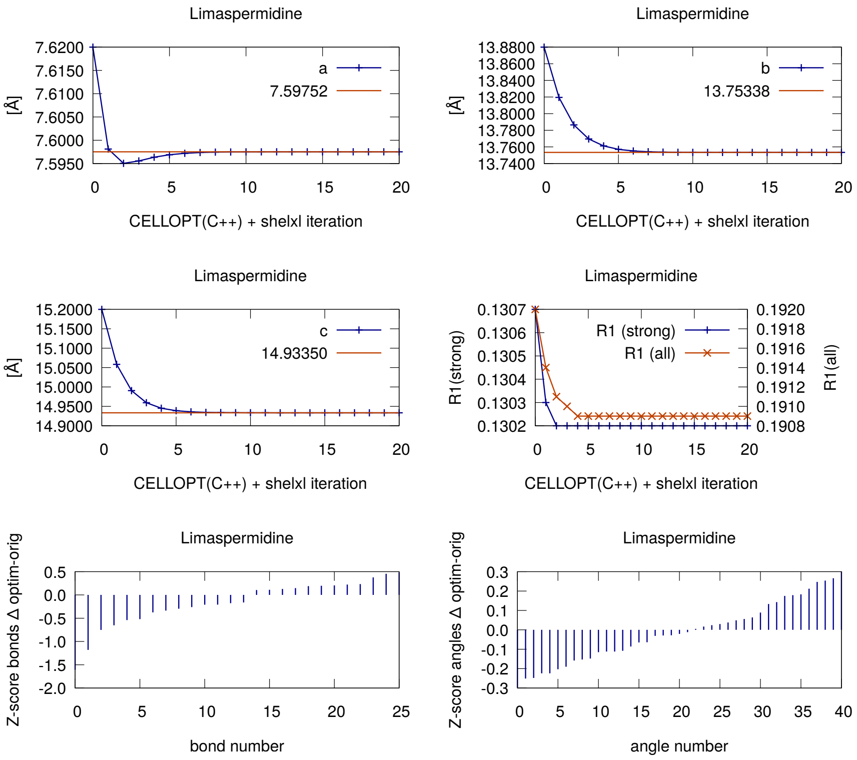

Fig. S8. Iterative cell optimisation of (+)-Limaspermidine. Orthorhombic space group: a, b, c-axes, R1 values. Z-scores show difference of the models after and before cell optimisation with CELLOPT(C++). Hence, negative values refer to a lower Z-score after optimisation and thus to an improvement of the geometry.

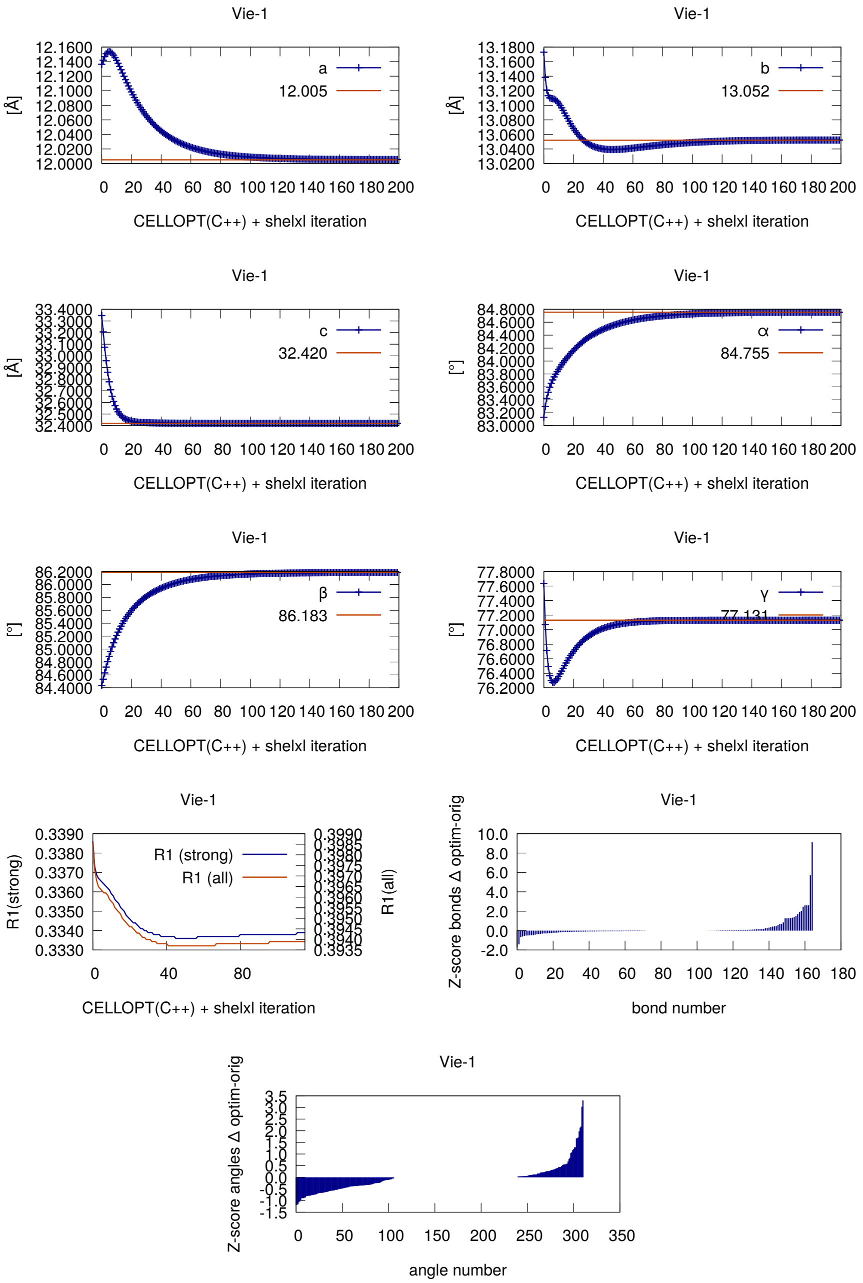

Fig. S9. Iterative cell optimisation of the Nd-MOF. Triclinic space group: a, b, c-axes, α-, β-, γ-angles, R1 values. Z-scores show difference of the models after and before cell optimisation with CELLOPT(C++). Hence, negative values refer to a lower Z-score after optimisation and thus to an improvement of the geometry.

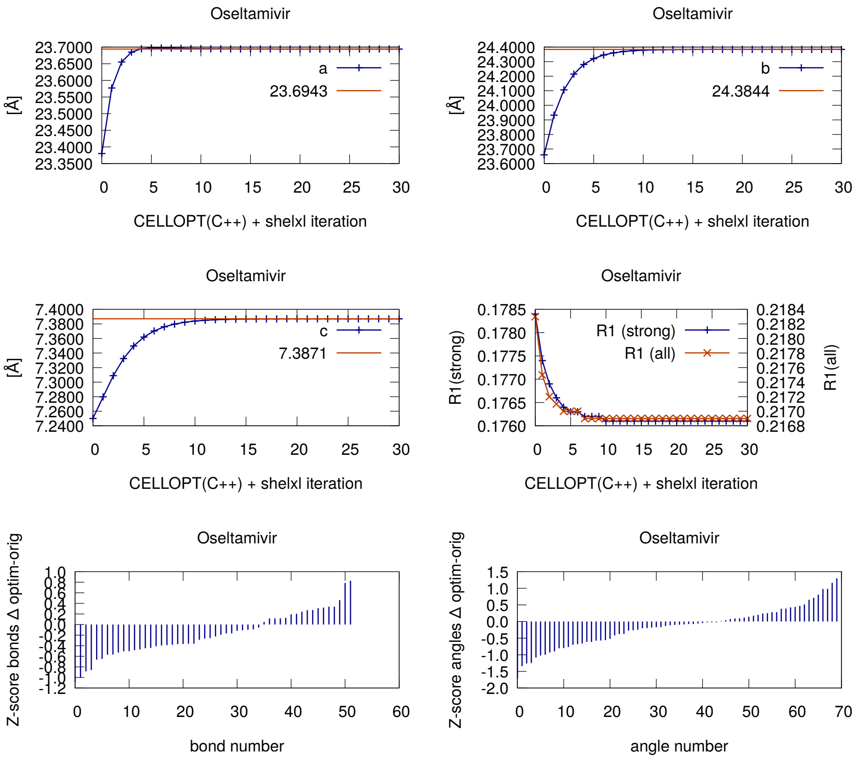

Fig. S10. Iterative cell optimisation of the Oseltamivir. Orthorhombic space group: a, b, c-axes, R1 values. Z-scores show difference of the models after and before cell optimisation with CELLOPT(C++). Hence, negative values refer to a lower Z-score after optimisation and thus to an improvement of the geometry.

4. Correction of optical distortions with XDS

4.1. Data processing work-flow including geometrical corrections

XDS has an in-built mechanism to correct for distortion of the detector plane (Kabsch, 2010). The look-up tables X-CORRECTIONS.cbf and Y-CORRECTIONS.cbf encode per-pixel shifts of the detector surface. The CORRECT step writes out GX-CORRECTIONS.cbf and GY-CORRECTIONS.cbf. These contain the pixel shifts based on the discrepancy between the calculated and the observed spot positions. This information can be applied by renaming GY-CORRECTIONS.cbf to Y-CORRECTIONS.cbf and GX-CORRECTIONS.cbf to X-CORRECTIONS.cbf respectively, just like the

recycling of improved experimental parameters by renaming GXPARMS.XDS to XPARMS.XDS. This way, optical distortions in the detector plane can be corrected for. In order to avoid parameter drifts from strong correlation between certain parameters, only the orientation of the unit cell axes, and the rotation axis were refined during the recycling of GX-CORRECTIONS.cbf and GY-CORRECTIONS.cbf.

1. Process all data in $P1$
2. Solve the structure, and refine using geometry restraints in SHELXL (DFIX/DANG)
3. Optimise unit cell constants with CELLOPT
4. Re-process data with the optimised cell and spacegroup
   (a) Fix cell, refine the detector distance once:
      - `JOB= CORRECT`
      - `REFINE(CORRECT)= ORIENTATION AXIS POSITION`
   (b) update the geometry (`ROTATION_AIXS`, `DETECTOR_DISTANCE`, `INCIDENT_BEAM_DIRECTION`, `ORGX`, `ORGY`) from `XDS_ASCII.HKL` to `XDS.INP`
   (c) Fix cell and detector distance and recycle GX-CORRECTIONS.cbf and GY-CORRECTIONS.cbf once.
      - `JOB= CORRECT`
      - `REFINE(CORRECT)= ORIENTATION AXIS`
   (d) Update `ROTATION_AXIS`, `ORGX`, and `ORGY` from `XDS_ASCII.HKL` to `XDS.INP`, similar to 4b
   (e) As last step, refine the unit cell parameters `REFINE(CORRECT)= ORIENTATION AXIS BEAM CELL` in spacegroup $P1$

When data resolution is sufficiently high, cell and position can be refined simultaneously in steps 4c and 4e.

References

Evans, P. (2006). *Acta Crystallogr,* D**62**, 72–82.

Evans, P. R. & Murshudov, G. N. (2013). *Acta Crystallogr,* D**69**, 1204–1214.

Kabsch, W. (2010). *Acta Crystallogr,* D**66**, 133–144.

Winn, M. D., Ballard, C. C., Cowtan, K. D., Dodson, E. J., Emsley, P., Evans, P. R., Keegan, R. M., Krissinel, E. B., Leslie, A. G. W., McCoy, A., McNicholas, S. J., Murshudov, G. N., Pannu, N. S., Potterton, E. A., Powell, H. R., Read, R. J., Vagin, A. & Wilson, K. S. (2011). *Acta Crystallogr,* D**67**, 235–242.
